# Effect of chronic delivery of the NOP/MOR partial agonist AT-201 and NOP antagonist J-113397 on heroin relapse in a rat model of opioid maintenance

**DOI:** 10.1007/s00213-024-06678-7

**Published:** 2024-09-13

**Authors:** Jennifer M. Bossert, Kiera E. Caldwell, Hannah Korah, Ashley Batista, Hannah Bonbrest, Ida Fredriksson, Shelley N. Jackson, Agnieszka Sulima, Kenner C. Rice, Nurulain T. Zaveri, Yavin Shaham

**Affiliations:** 1 Behavioral Neuroscience Branch, IRP/NIDA/NIH, Baltimore, MD U.S.A.; 2 Translational Analytical Core, IRP/NIDA/NIH, Baltimore, MD U.S.A.; 3 Molecular Targets and Medications Discovery Branch, IRP/NIDA, NIAAA/NIH, Baltimore, MD U.S.A.; 4 Astraea Therapeutics, 320 Logue Avenue, Mountain View, CA USA

**Keywords:** Opioid maintenance, Incubation of craving, Extinction, Context-induced reinstatement, Heroin self-administration, Reacquisition

## Abstract

**Rationale:**

The opioid crisis persists despite availability of effective opioid agonist maintenance treatments (methadone and buprenorphine). Thus, there is a need to advance novel medications for the treatment of opioid use and relapse.

**Objectives:**

We recently modeled maintenance treatment in rats and found that chronic delivery of buprenorphine and the mu opioid receptor (MOR) partial agonist TRV130 decreases relapse to oxycodone seeking and taking. In contrast, chronic delivery of the buprenorphine analog BU08028 had mixed effects on different heroin relapse-related measures. Here, we tested the effect of the mixed nociceptin (NOP) receptor/MOR partial agonist AT-201 and the NOP receptor antagonist J-113397 on different heroin relapse-related measures.

**Methods:**

We trained male and female rats to self-administer heroin (6-h/d, 14-d) in context A and then implanted osmotic minipumps containing AT-201 (0, 3.8, or 12 mg/kg/d) or J-113397 (0, 12.6, or 40 mg/kg/d). Next, we tested the effect of chronic delivery of the compounds on (1) incubation of heroin seeking in a non-drug context B, (2) extinction responding reinforced by heroin-associated discrete cues in context B, (3) context A-induced reinstatement of heroin seeking, and (4) reacquisition of heroin self-administration in context A.

**Results:**

In females, AT-201 modestly *increased* reacquisition of heroin self-administration and J-113397 modestly *decreased* incubation of heroin seeking. The compounds had no effect on the other relapse-related measures in females, and no effect on any of the measures in males.

**Conclusion:**

The NOP/MOR partial agonist AT-201 and the NOP antagonist J-113397 did not mimic buprenorphine’s inhibitory effects on relapse in a rat model of opioid maintenance treatment.

**Supplementary Information:**

The online version contains supplementary material available at 10.1007/s00213-024-06678-7.

## Introduction

Over the past decade, there has been a large increase in opioid-related deaths (Rudd et al. 2016; Hedegaard et al. 2017; Butelman et al. 2023). This ‘opioid crisis’ persists despite the availability of effective opioid agonist maintenance treatments, such as methadone and buprenorphine (Epstein et al. 2018; Skolnick 2018). This warrants a need to study and advance novel medications for the treatment of opioid use and relapse.

We recently combined a modified version of the ABA renewal model of context-induced reinstatement (Crombag and Shaham 2002; Bossert et al. 2019) with a rat model of opioid maintenance (Shaham et al. 1996; Sorge et al. 2005) to study the effect of novel opioid compounds on different relapse-related measures: extinction responding, context-induced reinstatement, and reacquisition of opioid self-administration, and time-dependent increases (incubation) of opioid seeking (Bossert et al. 2020, 2022).

We first established the predictive validity of our rat model using chronically delivered buprenorphine (via Alzet osmotic minipumps) and found that in both male and female rats, chronic buprenorphine decreased extinction responding, context-induced reinstatement, and reacquisition of oxycodone self-administration (Bossert et al. 2020). Next, we used the model to study the effect of two potential novel medications: the partial mu opioid receptor (MOR) agonist TRV130 (DeWire et al. 2013) and the buprenorphine analog BU08028 (Ding et al. 2016), a mixed partial agonist at MOR and nociceptin/orphanin FQ (N/OFQ) peptide (NOP) receptor (Bossert et al. 2020, 2022).

In the first study, we found that in oxycodone-trained male rats, chronic delivery of TRV130 mimicked buprenorphine’s effect on extinction responding, context-induced reinstatement, and reacquisition in male rats. In contrast, in female rats, chronic delivery of TRV130 only decreased extinction responding (Bossert et al. 2020). In the second study, we found that in heroin-trained male and female rats, chronic BU08028 delivery decreased incubation of heroin seeking in both sexes, decreased extinction responding in male rats only, had minimal effects on context-induced reinstatement of heroin seeking, but unexpectedly *increased* reacquisition in female rats (Bossert et al. 2022).

In the present study, we used our opioid maintenance relapse model to determine the effect of a different mixed partial agonist at NOP/MOR, AT-201 (formerly named SR16435) (Khroyan et al. 2011) on relapse-related behaviors in rats trained to self-administer heroin. Like buprenorphine, AT-201 decreases cocaine self-administration, an effect mediated by the combined activation of MOR and NOP receptors (Kallupi et al. 2018). Additionally, Ding et al. (2018) reported that in rhesus monkeys, acute pretreatment with the NOP/MOR partial agonist AT-121 (which has similar affinity and efficacy at NOP receptors but lower affinity and efficacy at MOR than AT-201) decreases oxycodone self-administration. Furthermore, AT-121 antinociceptive effects were like morphine but it produced less dependence and tolerance than morphine after repeated administration. Ding et al. (2018) also reported that AT-121 is not self-administered, suggesting low abuse liability.

We also tested in our opioid maintenance model the effect of the NOP receptor antagonist J-113397 (Ozaki et al. 2000a). We tested this compound because we found that AT-201 *increased* reacquisition of heroin self-administration in females and had minimal effects on the other relapse-related measures in males and females. Additionally, previous studies reported that in rats NOP receptor antagonists decrease alcohol self-administration and cue-induced reinstatement (Rorick-Kehn et al. 2016; Kallupi et al. 2017; Borruto et al. 2021). Furthermore, genetic deletion of the rat NOP receptor decreases alcohol and heroin self-administration under fixed-ratio and progressive-ratio reinforcement schedules (Kallupi et al. 2017).

## Methods

### Subjects

We used male (*n* = 73) and female (*n* = 91) Sprague–Dawley rats (Charles River) weighing 250–350 g (males) or 175–225 g (females) before surgery. We maintained the rats under a reverse 12:12 h light/dark cycle (lights off at 8:00 a.m.) with food and water freely available. We housed two rats/cage prior to surgery and either two rats/cage (food self-administration rats) or individually (heroin self-administration rats) after surgery. We excluded 5 rats due to health problems during the experiment (*n* = 4, 3 males, 1 female) or failure to acquire heroin self-administration (*n* = 1, female) during training. We performed the experiments in accordance with the NIH Guide for the Care and Use of Laboratory Animals (8th edition) under protocols approved by the Animal Care and Use Committee of the NIDA IRP.

### Drugs

We received heroin hydrochloride (HCl) from the NIDA pharmacy and dissolved it in sterile saline. We chose a unit dose of 0.1 and 0.05 mg/kg/infusion for self-administration training based on our previous work (Bossert et al. 2004, 2020, 2022). N. Zaveri provided us with AT-201 as a hydrochloride salt and we dissolved the compound in 50% sterile dimethyl sulfoxide (DMSO) in 30% 2-hydroxypropyl beta-cyclodextrin in sterile water at concentrations that yielded chronic delivery of 3.8 or 12 mg/kg/day. A. Sulima and K. Rice provided us with J-113397 as a free base and we dissolved the compound in a solution of 50% sterile DMSO and 50% ethanol (EtOH) at concentrations that yielded chronic delivery of doses of 12.6 or 40 mg/kg/day. The vehicles for the compounds were based on solubility and ability to keep the compounds stable in solution in a 37-degree incubator for 3 weeks. We chose the chronic doses of the compounds based on acute doses used in other studies (Recker and Higgins 2004; Khroyan et al. 2009; Kallupi et al. 2018). The investigators were not blind to the minipump dose conditions.

### Surgery

Intravenous surgery: We anesthetized the rats with isoflurane (5% induction; 2–3% maintenance, Covetrus). We attached silastic catheters to a modified 22-gauge cannula cemented to polypropylene mesh (Amazon or Industrial Netting), inserted the catheter into the jugular vein, and fixed the mesh to the mid-scapular region of the rat (Caprioli et al. 2015; Bossert et al. 2022). We injected the rats with ketoprofen (2.5 mg/kg, s.c., Covetrus) during surgery and on the following day to relieve pain and decrease inflammation. Rats recovered for 6–8 days before heroin self-administration training. During all experimental phases, we flushed the catheters daily with gentamicin in sterile saline (4.25–5 mg/mL, 0.1 mL, Fresenius Kabi or Covetrus).

Minipump surgery: Chronic delivery of AT-201 and J-113397 was achieved by implanting osmotic minipumps subcutaneously (Alzet model 2ML2, 5 μl/h for 14–16 days, Durect Corporation) as described in our previous studies (Bossert et al. 2020, 2022). We anesthetized the rats with isoflurane as described above, injected them with ketoprofen, and made a small incision mid-scapular (food experiments) or on the right side of the intravenous backmount (heroin experiments). We used a hemostat to spread apart the subcutaneous connective tissue to make a small pocket for the pump. We placed the osmotic pumps into the pocket with the flow moderator directed away from the incision. We then closed the incisions with sterile surgical suture or wound clips.

### Apparatus

We trained and tested the rats in standard Med Associates self-administration chambers. Each chamber had two levers located 7.5–8 cm above the grid floor on opposing walls. Lever presses on the active, retractable lever activated pellet dispenser or the infusion pump, whereas lever presses on the inactive, non-retractable lever had no programmed consequences. As in our previous studies (Bossert et al. 2019, 2020, 2022), the active lever was inserted into the chamber at the start of the self-administration and test sessions and retracted at the end of these sessions, while the inactive lever was constantly present in the chamber. For Exp. 2 & 3, the two contexts differed in their auditory, visual, and tactile cues, as in our previous studies (Bossert et al. 2019, 2020, 2022). We refer to the contexts as A and B, where A is the context for self-administration training and reacquisition, and B is the context for extinction. We counterbalanced the physical environments of contexts A and B.

### Food self-administration (Exp. 1)

**Fig. 1:**
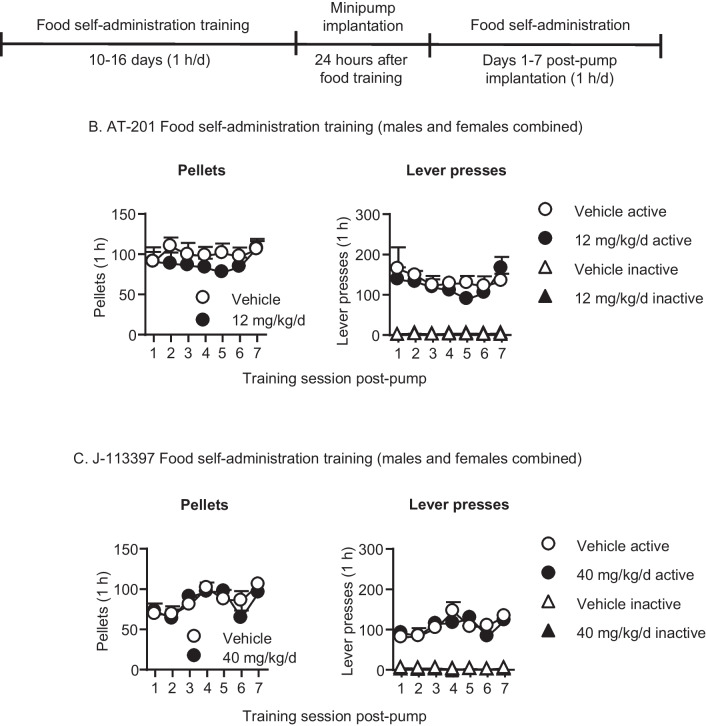
Effect of chronic delivery of AT-201 and J-113397 on food self-administration in male and female rats. **A** Timeline of Exp. 1. **B** AT-201: Number of pellets consumed and inactive and active lever presses after the rats were implanted with Alzet osmotic minipumps containing vehicle (*n* = 6, 4 males, 2 females) or 12 mg/kg/day AT-201 (*n* = 6, 3 males, 3 females). **C** J-113397: Number of pellets consumed and inactive and active lever presses after the rats were implanted with Alzet osmotic minipumps containing vehicle (*n* = 6, 2 males, 4 females) or 40 mg/kg/day J-113397 (*n* = 7, 3 males, 4 females). All data are mean ± SEM

The timeline for Exp. 1 is shown in Fig. 1A. The goal of Experiment 1 was to rule out sedative or other non-specific effects as explanations for potential relapse-related effects of AT-201 or J-113397. For this purpose, we tested the effect of the higher doses of the compounds (12 mg/kg/day for AT-201 and 40 mg/kg/day for J-113397, used in Exp. 2–3) on high-rate food-maintained operant responding in previously drug-naïve male and female rats.

The experiment consisted of three phases: food self-administration training (10–16 days, 1 h/d), minipump-implantation surgery, and then continuance of food self-administration (7 days, 1 h/d). We mildly food-restricted the rats for the first 3–5 days of training (food was removed around 8 am; rats were trained between 1 and 5 pm). Three rats from AT-201 were subsequently food restricted overnight (one night) to speed up food self-administration acquisition. Each session began with the illumination of a houselight that remained on for the entire session; the active lever was inserted into the chamber 10 s after the houselight was illuminated. On the first day, we gave the rats a 1-h magazine-training session during which 1 pellet was delivered noncontingently every 2 min. During the self-administration sessions, lever presses under the FR1, 20-s timeout reinforcement schedule led to the delivery of one 45-mg pellet of palatable food (TestDiet, Cat # 1,811,155, 12.7% fat, 66.7% carbohydrate, and 20.6% protein) (Calu et al. 2014); pellet deliveries were paired with a 20-s white-light cue. At the end of the session, the white light was turned off and the active lever was retracted. We gave the rats 10–16 days of food self-administration training prior to the minipump-implantation surgery. We then implanted the minipumps with vehicle (50% sterile DMSO in 30% 2-Hydroxypropyl Beta-cyclodextrin in sterile water [vehicle for AT-201] or 50% sterile DMSO and 50% EtOH [vehicle for J-113397]) or 12 mg/kg/day (AT-201, *n* = 6/dose) or 40 mg/kg/day (J-113397, *n* = 6–7/dose). We ran the rats in food self-administration sessions for 7 consecutive days thereafter. We matched the rats in the different dose groups for total pellet intake during food self-administration training.

### Heroin self-administration (Exp. 2–3)

**Fig. 2:**
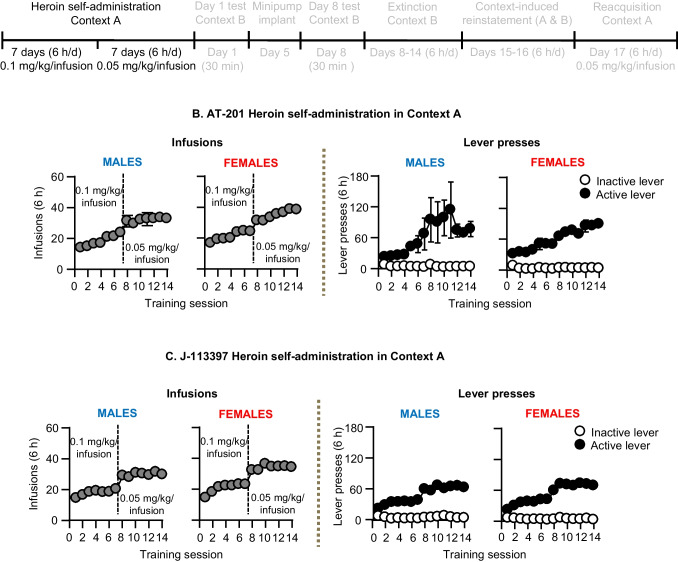
Heroin self-administration training in Context A. **A** Timeline of Exp. 2–3. **B** AT-201: Number of heroin infusions and lever presses during heroin self-administration in Context A (*n* = 53, 24 males, 29 females). **C** J-113397: Number of heroin infusions and lever presses during heroin self-administration in Context A (*n* = 54, 23 males, 31 females). All data are mean ± SEM

Training in context A (14 days): The timeline for heroin training for Exp. 2 & 3 are shown in Fig. 2A. We trained the rats to self-administer heroin HCl in context A for 6 h/day (six 1-h sessions separated by 10 min) for 14 days. Each session began with the illumination of a houselight that remained on for the entire session; the active lever was inserted into the chamber 10 s after the houselight was illuminated. During training, the rats earned heroin infusions by pressing on the active lever; infusions were paired with a compound tone–light cue for 3.5 s under an FR1 reinforcement schedule with a 20-s timeout after each infusion. Heroin was infused at a volume of 100 µl over 3.5 s at a dose of 0.10 mg/kg/infusion (the first 7 sessions) and then 0.05 mg/kg/infusion (the last 7 sessions). Responses on the active lever during the timeout period were recorded but did not result in heroin infusions. Presses on the inactive lever were recorded but had no programmed consequences. At the end of each session, the houselight was turned off and the active lever was retracted. If we suspected catheter failure during training, we tested patency with Diprivan (propofol, NIDA pharmacy, 10 mg/mL, 0.1–0.2 mL injection volume, i.v.). If the catheter was not patent, we catheterized the left jugular vein. We implanted the minipumps 5 days after training (see below for details).

### Experiments 2–3: Effect of chronic AT-201 and J-113397 on incubation of heroin seeking, extinction responding, context-induced reinstatement, and reacquisition

**Fig. 3:**
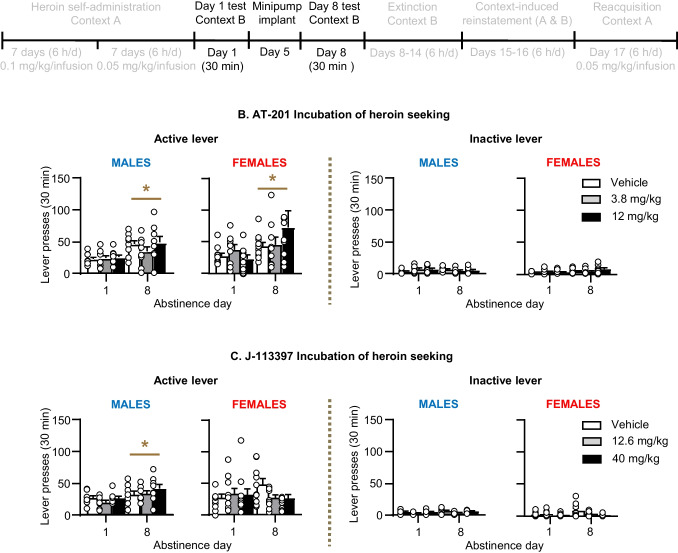
Effect of chronic delivery of AT-201 and J-113397 on incubation of heroin seeking in Context B. **A** Timeline of Exp. 2–3. **B** AT-201: Number of active lever presses and inactive lever presses on abstinence day 1 (before minipump implantation) and on abstinence day 8 (after minipump implantation) in context B. Vehicle (*n* = 18, 8 males, 10 females), 3.8 mg/kg/day (*n* = 17, 8 males, 9 females), and 12 mg/kg/day AT-201 (*n* = 18, 8 males, 10 females). **C** J-113397: Number of active lever presses and inactive lever presses on abstinence day 1 (before minipump implantation) and on abstinence day 8 (after minipump implantation) in context B. Vehicle (*n* = 18, 8 males, 10 females), 12.6 mg/kg/day (*n* = 17, 7 males, 10 females), and 40 mg/kg/day J-113397 (*n* = 19, 8 males, 11 females). During the 30 min extinction sessions, active lever presses led to contingent presentations of the tone-light clue previously paired with heroin, but not heroin. All data are mean ± SEM. * Different from Day 1, p < 0.05

**Fig. 4:**
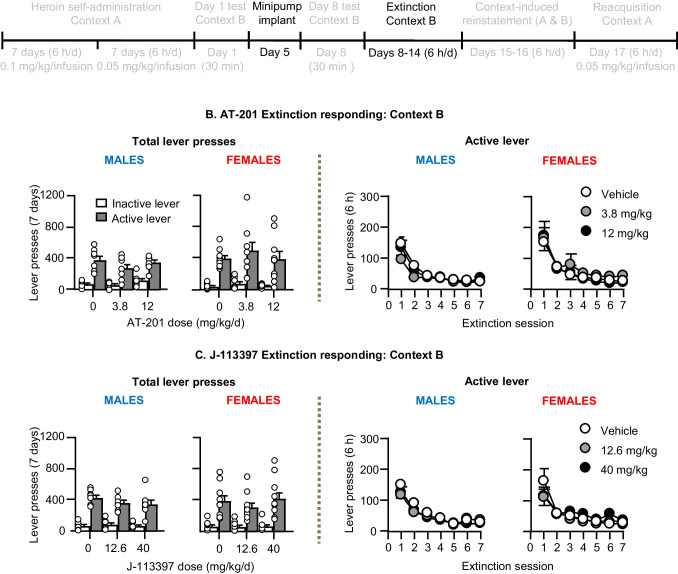
Effect of chronic delivery of AT-201 and J-113397 on extinction responding in Context B. **A** Timeline of Exp. 2–3. **B** AT-201: Number of active and inactive lever presses and number of active lever presses during the seven 6-h extinction sessions in context B. Vehicle (*n* = 18, 8 males, 10 females), 3.8 mg/kg/day (*n* = 17, 8 males, 9 females), and 12 mg/kg/day AT-201 (*n* = 17, 7 males, 10 females). **C** J-113397: Number of active and inactive lever presses and number of active lever presses during the seven 6-h extinction sessions in context B. Vehicle (*n* = 18, 8 males, 10 females), 12.6 mg/kg/day (*n* = 17, 7 males, 10 females), and 40 mg/kg/day J-113397 (*n* = 19, 8 males, 11 females). During the 6-h extinction sessions, active lever presses led to contingent presentations of the tone-light clue previously paired with heroin, but not heroin. All data are mean ± SEM

**Fig. 5:**
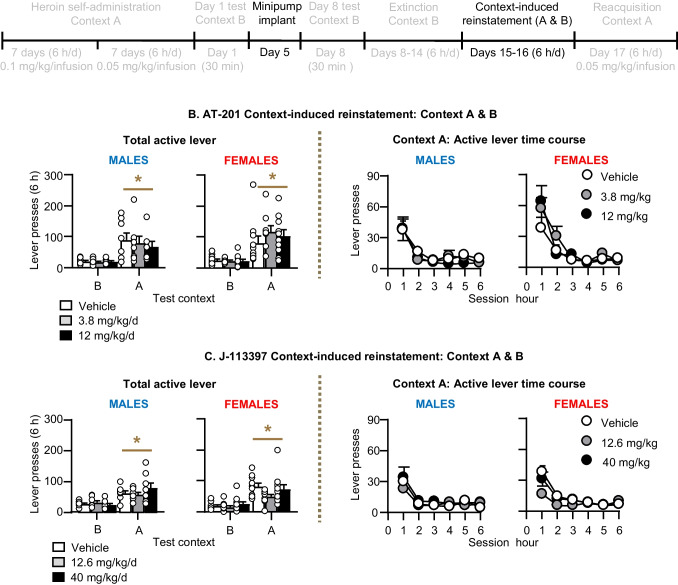
Effect of chronic delivery of AT-201and J-113397 on context-induced reinstatement in Context A. **A** Timeline of Exp. 2–3. **B** AT-201: Number of active lever presses during the 6-h reinstatement tests in contexts A & B and number of active lever presses during the six 1-h sessions in context A. Vehicle (*n* = 18, 8 males, 10 females), 3.8 mg/kg/day (*n* = 17, 8 males, 9 females), and 12 mg/kg/day AT-201 (*n* = 17, 7 males, 10 females **C** J-113397: Number of active lever presses during the 6-h reinstatement tests in contexts A & B and number of active lever presses during the six 1-h sessions in context A. Vehicle (*n* = 18, 8 males, 10 females), 12.6 mg/kg/day (*n* = 17, 7 males, 10 females), and 40 mg/kg/day J-113397 (*n* = 19, 8 males, 11 females). During the context-induce reinstatement tests, active lever presses led to contingent presentations of the tone-light clue previously paired with heroin, but not heroin. All data are mean ± SEM. * Different from context B, p < 0.05

The timelines for Exp. 2–3 are shown in Figs. 3A, 4A, 5A, & 6A. The goal of these experiments was to test the effect of chronic delivery of AT-201 and J-113397 on our relapse-related measures. Prior to the relapse tests, we trained the rats to self-administer heroin in context A for 6 h per day for 14 days as described above. Next, after a short 30-min extinction session on abstinence day 1 (see below), we implanted the rats with minipumps on abstinence day 5. For Exp. 2, we implanted the rats with minipumps containing vehicle (8 males, 10 females), 3.8 mg/kg/d (8 males, 9 females), or 12 mg/kg/d (8 males, 10 females) AT-201. For Exp. 3, we implanted the rats with minipumps containing vehicle (8 males, 10 females), J-113397 12.6 mg/kg/d (7 males, 10 females), or 40 mg/kg/d (8 males, 11 females). We matched the rats in the different dose groups for total heroin infusions during heroin self-administration training and for extinction responding in context B on abstinence day 1.

### Incubation of heroin seeking in context B (abstinence days 1 and 8)

We tested rats in a brief (30 min) extinction session in context B one day after the last day of heroin self-administration training (abstinence day 1). During the test session, responses on the previously active lever led to presentations of the tone-light cue but not heroin infusions. On abstinence day 5, we implanted the rats with Alzet minipumps containing vehicle or AT-201 (3.8 or 12 mg/kg/day) or J-113397 (12.6 or 40 mg/kg/day). We used the first 30 min of the 6-h extinction session on abstinence day 8 to evaluate whether chronic AT-201 or J-113397 delivery would decrease incubation of heroin seeking. In previous studies, we observed reliable incubation of heroin seeking in context A and incubation of methamphetamine seeking in context B after one week of abstinence (Shalev et al. 2001; Adhikary et al. 2017).

### Extinction responding in context B (abstinence days 8–14)

We ran the rats under extinction conditions in context B for 6 h per day (six 1-h sessions separated by 10 min) for 7 days. During this phase, responses on the previously active lever led to presentation of the discrete tone-light cue but not heroin infusions.

### Context-induced reinstatement in contexts A and B (abstinence days 15–16)

We tested the rats under extinction conditions (see above) for 6 h per day for 2 days in context A and context B in a counterbalanced order. We excluded one male rat’s data (AT-201) from the context tests only due to being a statistical outlier (951 lever presses in 3 h in context A, more than 3 standard deviations above the group mean).

### Reacquisition of heroin self-administration in context A (abstinence day 17)

We tested reacquisition of heroin self-administration during one 6-h session in context A. During testing, lever presses were reinforced by heroin (0.05 mg/kg/infusion, FR1 reinforcement schedule with a 20-s timeout after each infusion) and the discrete tone-light cue. After the 6-h session, we tested catheter patency with propofol (NIDA pharmacy, 10 mg/mL, 0.1–0.2 mL injection volume, i.v.). We excluded 4 rat’s data (J-113397, two vehicle and two 40 mg/kg/day, all females) from the reacquisition test only because they did not demonstrate an immediate anesthetic response after propofol injections.

### Naloxone-precipitated withdrawal

We measured naloxone-precipitated withdrawal (Cerletti et al. 1976) to see if chronic delivery of either AT-201 or J-113397 produced MOR-related withdrawal symptoms. For AT-201, we tested 6 rats (3 males, 3 females, all 12 mg/kg/day) at the end of the AT-201 food self-administration experiment and 4 rats (1 male, 3 females, all 12 mg/kg/day) at the end of one of the AT-201 heroin experiments. For J-113397, we tested 2 female rats from a pilot J-113397 food study (these rats were also subsequently used to measure plasma level of the drug, see below) and 7 rats (3 males, 4 females, all 40 mg/kg/day) at the end of the J-113397 food self-administration experiment. We weighed each rat and then placed them individually in a large (13.03 L × 6.26 W × 5.19 H in) clear cage with standard bedding for 5 min to acclimate to the cage. We then injected each rat with saline (1 ml/kg, s.c.) and placed the rat back in the cage and manually scored behaviors (see below) for 15 min. We then weighed each rat again, injected them with naloxone HCl (1 mg/kg, s.c.; dissolved in saline, 1 mL/kg, Tocris, Cat #0599) and again manually scored behaviors for 15 min. At the end, we weighed each rat again and placed them back into their homecage. The behaviors scored were: 1) teeth chattering (chattering/grinding of teeth, lip smacking), 2) excessive grooming scratching/rubbing fur, repeated paw movements over face), 3) wet dog shakes (head and entire body shaking repeatedly), 4) hyperirritability upon touch (10 times in a row), 5) number of dry fecal matter, and 6) diarrhea (present or absent) (Blasig et al. 1973). We totaled the number of withdrawal signs and compared the saline score to the naloxone score. We chose raters experienced with measuring naloxone-precipitated withdrawal that were blind to the minipump conditions.

### Measurement of plasma J-113397 levels

We took plasma samples from 10 rats used in a food pilot study (data not shown). We trained rats to self-administer palatable food pellets (see Exp. 1 below) and then implanted them with vehicle (70% DMSO, 30% EtOH; *n* = 4), or J-113397 at doses of 10 mg/kg/day (*n* = 2), 20 mg/kg/day (*n* = 2) or 40 mg/kg/day (*n* = 2). Food self-administration training resumed the day after the minipump implantation. Plasma samples were processed for the mass spectrometry identification of J-113397 10–15 days after minipump implantation. We anesthetized the rats with isoflurane before decapitation. We collected the blood into 1.5 mL Eppendorf tubes containing 50 µL of 1000 IU/mL heparin stored on wet ice. We then briefly microfuged the tubes and returned them to the wet ice for about 30 min. We then transferred the tubes to a 4-degree centrifuge at 1500 rotations per minute (rpm) for 10 min, and then pipetted the plasma into new tubes and stored them at -80 degrees until analysis.

For extraction, we protein precipitated 50 µL of plasma with 300 µL of acetonitrile. The samples were vortexed for 5 s, shaken for 10 min at 2,000 rpm with a ThermoMixer C (Eppendorf) and centrifuged at 13,000 rpm for 10 min at 4 °C in a Sorvall ST 40R centrifuge (ThermoFisher). We collected 200 µL of supernatant into a glass sample vial. LC–MS analysis was conducted using a Vanquish UHPLC system (ThermoFisher) with tandem Orbitrap Exploris 120 mass spectrometer (ThermoFisher). Reverse phase chromatography was performed using an Accucore Biphenyl column, 2.1 × 50 mm, 2.6 µm particle size column (ThermoFisher) with 0.1% formic acid as mobile phase A and acetonitrile as mobile phase B. The flow rate was held at 0.4 mL/min for the 6-min run with a sample injection volume of 5µL. The solvent gradient for mobile phase B was as follows: 0-to-0.5 min held at 10%, 0.5-to-2.5 min increased from 10 to 98%, from 2.5-to-4.5 min held at 98%, 4.5-to-5.5 min decreased from 98 to 10%, and then held at 10% to 6 min. Analysis was performed in positive ion mode with a heated electron spray ionization (HESI) source, full scan mass range of 300 – 600 m/z, mass accuracy of 5 ppm and Orbitrap mass resolution setting of 120,000. Stock solutions of J-113397 in DMSO were used to spike blank plasma at calibration standards of 2.5, 5, 10, 25, 50, 100, 250, 500, 1,000 ng/mL. XCalibur v 4.4.16.14 (ThermoFisher) software was used to integrate and report peak area for the M + H ion of J-113397 (400.2959 m*/z* at a retention time of 3.7 min) and to plot and fit a standard curve and to interpolate unknown values. The quantitative assay range of the standard curve was 2.5 to 1000 ng/mL.

### Statistical analysis

We analyzed the data with repeated-measures or univariate ANOVA using SPSS (Version 27, GLM procedure); which are also described in the Supplemental Table S1. For naloxone-precipitated withdrawal, we performed t-tests to compare baseline withdrawal scores to naloxone withdrawal scores and to compare baseline body weight with body weight after naloxone injections. Because our ANOVAs yielded multiple main and interaction effects, we report only effects that are critical for data interpretation. For complete statistical results, see Table S1.

## Results

### Naloxone-precipitated withdrawal

*AT-201:* Naloxone injections (1 mg/kg, s.c.) in rats implanted with minipumps containing AT-201 (12 mg/kg/d) produced signs of opioid withdrawal (Table S2). The mean ± SEM withdrawal score after saline injections (baseline) was 6.1 ± 1.5 and after naloxone injections was 26.4 ± 3.9 (t_9_ = 4.7, p = 0.001). The most prominent withdrawal signs were teeth chattering, number of dry fecal matter/diarrhea, and body weight loss (7.6 ± 0.9 g during the 15-min test, Table S2).

*J-113397:* Naloxone injections (1 mg/kg, s.c.) in rats implanted with minipumps containing J-113397 (40 mg/kg/d) produced some eye blinking and lip-smacking but no clear signs of opioid withdrawal. The mean ± SEM withdrawal score after saline injections was 0.7 ± 0.4 and after naloxone injections was 2.1 ± 0.9 (t_8_ = 1.0, p = 0.35). Naloxone injections did not produce dry fecal matter, diarrhea, or body weight loss (0.3 ± 0.5 g during the 15-min test, Table S2).

### Measurement of plasma J-113397 levels

We used an LC–MS assay to measure plasma levels of different J-113397 doses. We analyzed samples from four groups (*n* = 2–4 per dose): vehicle, 10 mg/kg/day, 20 mg/kg/day, and 40 mg/kg/day. We did not detect J-113397 in the vehicle group. The concentration (ng/mL, mean ± SEM) of J-113397 in plasma were increased in a dose-dependent manner: 10 mg/kg/day = 16.6 ± 2.25; 20 mg/kg/day = 85.6 ± 5.6; and 40 mg/kg/day = 135.8 ± 58.2 (Table S3).

### Exp. 1: Effect of chronic delivery of AT-201 and J-113397 on food self-administration (Fig. 1)

Chronic delivery of AT-201 and J-113397 had no effect on food-reinforced responding after minipump surgery. For AT-201, there was a significant main effect of Lever (F_1,10_ = 99.5, p < 0.001); no other main effects or interactions were significant for Pellets or Lever (p > 0.05). (Table S1). For J-113397, there was a significant main effect of Session (F_6,66_ = 14.6, p < 0.001) and interaction of J-113397 Dose × Session (F_6,60_ = 2.5, p = 0.032) for pellets. For lever presses, there were significant main effects of Lever (F_1,11_ = 156.8, p < 0.001) and Session (F_6,66_ = 7.9, p < 0.001), and significant interactions for Lever × Session (F_6,66_ = 8.2, p < 0.001) and Lever × Session × J-113397 Dose (F_6,66_ = 2.5, p = 0.033), but no main effects of J-113397 Dose (p values > 0.05). These significant interactions with J-113397 Dose are due to the more fluctuating responding over time in rats implanted with minipumps containing J-113397 than in rats implanted with minipumps containing the vehicle.

### Exp. 2–3: Heroin self-administration training (Fig. 2).

In Exp. 2–3, we trained the rats to self-administer heroin at 0.1 mg/kg/infusion for the first 7 days, followed by 0.05 mg/kg/infusion for the next 7 days. Rats of both sexes demonstrated reliable heroin self-administration as indicated by an increase in the number of heroin infusions and active lever presses over days, and a compensatory increase in the number of infusions earned when we halved the dose (Fig. 2 and Table S1 for statistics). There were no significant sex differences in heroin self-administration (Table S1).

### Exp. 2–3: Effect of chronic delivery of AT-201 and J-113397 on the relapse-related measures

#### Incubation of heroin seeking in Context B (Fig. 3)

Active lever presses in the vehicle condition were higher on abstinence day 8 than on day 1 (incubation of heroin seeking). Chronic delivery of AT-201 did not significantly decrease heroin seeking on abstinence day 8 in male or female rats. Chronic delivery of J-113397 did not decrease incubation of heroin seeking in male rats but modestly decreased incubation in female rats (see below). The initial ANOVAs, which included the between-subjects factors of Sex (males, females) and Dose (AT-201 [0, 3.8, 12 mg/kg/day] or J-113397 [0, 12.6, 40 mg/kg/day]) and the within-subjects factors of Lever (inactive, active) and Day (1, 8) showed significant effects of Lever and Day, but not Sex or Dose (see (Table S1 for statistical results). However, a visual inspection of the female data in Fig. 3C suggests that both doses of J-113397 decreased incubation. Indeed, one-way ANOVA of the active lever data showed a significant effect of J-113397 Dose (F_2,27_ = 5.5, p = 0.01) and post-hoc Fisher PLSD tests showed significant effects between the vehicle condition vs. the 12.6 mg/kg/day dose (p = 0.023) and the 40 mg/kg/dose (p = 0.004).

#### Extinction responding in Context B (Fig. 4)

Chronic delivery of AT-201 or J-113397 had no effect on active lever presses reinforced by the discrete tone + light cue in context B (extinction responding, 7 days, 6-h/d). The ANOVA, which included the between-subjects factors of Sex and Dose (AT-201 [0, 3.8, 12 mg/kg/day] or J-113397 [0, 12.6, 40 mg/kg/day]) and the within-subjects factors of Lever and Session (1–7) showed significant effects of Lever and Session, but not Sex or Dose (see (Table S1 for statistical results).

#### Context-induced reinstatement (Fig. 5)

Active lever presses in Context A were higher than those in Context B (context-induced reinstatement) in both sexes. Chronic delivery of AT-201 or J-113397 had no effect on context-induced reinstatement of heroin seeking in either sex. The ANOVA, which included the between-subjects factors of Sex and Dose (AT-201 [0, 3.8, 12 mg/kg/day] or J-113397 [0, 12.6, 40 mg/kg/day]) and the within-subjects factors of Lever and Context (B,A) showed significant effects of Lever and Context, but not Sex or Dose (see (Table S1 for statistical results).

#### Reacquisition in Context A (Fig. 6)

**Fig. 6:**
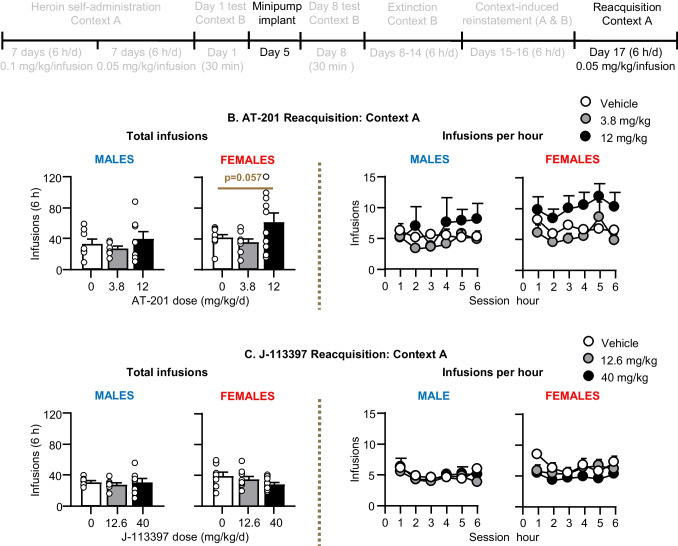
Effect of chronic delivery of AT-201 and J-113397 on reacquisition in Context A. **A** Timeline of Exp. 2–3. **B** AT-201: Number of heroin infusions (0.05 mg/kg/infusion) during the 6-h reacquisition test in context A and number of heroin infusions per hour. Vehicle (*n* = 18, 8 males, 10 females), 3.8 mg/kg/day (*n* = 17, 8 males, 9 females), and 12 mg/kg/day AT-201 (*n* = 18, 8 males, 10 females). **C** J-113397: Number of heroin infusions (0.05 mg/kg/infusion) during the 6-h reacquisition test in context A and number of heroin infusions per hour. Vehicle (*n* = 16, 8 males, 8 females), 12.6 mg/kg/day (*n* = 17, 7 males, 10 females), and 40 mg/kg/day J-113397 (*n* = 17, 8 males, 9 females). All data are mean ± SEM

Chronic delivery of AT-201 had no significant effect on reacquisition of heroin self-administration in male rats, but *increased* reacquisition in female rats. The ANOVA for number of infusions, which included the between-subjects factors of Sex and AT-201 Dose (0, 3.8, 12 mg/kg/day) and the within-subjects factor Hour (1–6) showed significant effects of Sex (F_1,47_ = 4.4, p = 0.041), AT-201 Dose (F_2,47_ = 3.6, p = 0.034), and Hour (F_5,235_ = 2.3, p = 0.043). Subsequent analyses within each sex showed a significant effect of Hour (F_5,130_ = 3.2, p = 0.010) and a trend for AT-201 Dose (F_2,26_ = 3.2, p = 0.057) for female but not male rats (p values > 0.1). Initial analysis of the J-113397 data using the factors of Sex, J-113397 Dose, and Hour did not show significant main or interaction effects on reacquisition of heroin self-administration in male or female rats. However, in females both doses of J-113397 decreased heroin intake in the first hour (p = 0.042 and 0.026 for vehicle vs. 12.6 and 40 mg/kg/day, respectively, Fig. 6C).

The results of Exp. 2–3 show that chronic delivery of AT-201 and J-113397 resulted in somewhat sex-dependent effects on the relapse-related measures, with J113397 modestly decreasing incubation of heroin seeking and AT-201 modestly increasing reacquisition of heroin self-administration selectively in female rats. There were no significant effects of the two compounds any of the relapse-related measures in male rats.

## Discussion

We used our rat model of opioid maintenance to test the effect of chronic delivery of the NOP/MOR partial agonist AT-201 and the NOP receptor antagonist J-113397 on relapse-related measures of heroin seeking and taking in rats. In males, chronic AT-201 or J-113397 delivery had no effect on any of the relapse measures. In females, AT-201 modestly *increased* reacquisition of heroin self-administration and J-113397 modestly *decreased* incubation of heroin seeking. These modest effects of AT-201 and J-113397 should be interpreted with caution and require independent replication, because they are based on exploratory post-hoc analyses after the formal factorial ANOVA did not show AT-201 or J-113397 Dose by Sex interaction. Furthermore, our sample sizes (*n* = 7–11 per sex in the different experimental conditions) may not be sufficiently large enough to reliably detect sex differences.

### Effects of AT-201 and J-113397 on heroin relapse

The largely negative data with AT-201 in the opioid maintenance rat model agree with our previous findings with the buprenorphine analog BU08028 (Bossert et al. 2022), which has similar affinity and efficacy at MOR and NOP to AT-201 (Khroyan et al. 2011). Additionally, like BU08028, AT-201 appears to *increase* reacquisition of heroin self-administration selectively in females. These results are different from those of buprenorphine, which decreases opioid seeking and taking in both sexes (Bossert et al. 2020).

The reasons for the different effects of buprenorphine vs. BU08028 and AT-201, which have similar partial agonist action at MOR (Jaffe 1990; Khroyan et al. 2011), are unknown. One possibility is that buprenorphine acts as an antagonist at the kappa opioid receptor (KOR) (Jaffe 1990), while BU08028 and AT-201 do not. This possibility is unlikely because we previously found that chronic blockade of KOR with nor-BNI (Portoghese et al. 1987) has no effect on extinction, context-induced reinstatement, and reacquisition of oxycodone self-administration (Bossert et al. 2020).

Another explanation for the different effects of buprenorphine vs. BU08028 and AT-201 is their different agonist efficacies at NOP receptors. Buprenorphine has low affinity and efficacy, whereas BU08028 and AT-201 have higher affinity (Khroyan et al. 2007, 2011; Ding et al. 2016). Additionally, unlike buprenorphine, which has higher potency as a MOR agonist, AT-201 has higher potency as a NOP agonist, but lower potency at MOR than buprenorphine or BU08028. We speculate that under our experimental conditions, agonist activity at NOP counteracted the MOR agonist-mediated inhibitory effect on the relapse-related measures.

Indirect evidence for this speculation comes from previous studies showing that NOP agonist activation decreases MOR-mediated antinociception induced by mixed NOP/MOR agonists such as buprenorphine and AT-201 (Khroyan et al. 2009). The speculation that activation of NOP receptors decreases MOR agonist-mediated effects is further supported by studies showing that the antinociceptive effects of buprenorphine are increased in NOP receptor knockout mice, an effect mimicked by pretreatment with J-113397 in wild-type but not NOP knockout mice (Lutfy et al. 2003; Yamamoto et al. 2006). Additionally, intra-ventricular injections of nociceptin blocked morphine conditioned place preference (Ciccocioppo et al. 2000), indicating that activation of NOP receptors decreases opioids’ rewarding effects. However, the recent report that cebranopadol, a potent receptor agonist at both NOP and MOR (Dasgupta et al. 2022) decreases heroin self-administration and yohimbine-induced reinstatement (Cannella et al. 2024) is inconsistent with our speculation.

The negative results of AT-201 on the heroin relapse-related measures are different from previous studies in non-human primates on the inhibitory effect of the bifunctional NOP/MOR partial agonist AT-121 on oxycodone self-administration (Ding et al. 2018) as well as the rat study of Cannella et al. described above. One potential reason for the different results is that we used chronic minipump delivery of AT-201, while in Ding et al. (2018) and Cannella et al. (2024) studies, the mixed MOR/NOP agonists were injected acutely. Chronic and acute administration of MOR/NOP agonists may cause different intracellular effects on MOR and NOP receptors, including desensitization of MORs by NOP activation (Mandyam et al. 2002). Another potential reason for the different results is that we used different relapse-related measures, whereas in the studies of Ding et al. (2018) and Cannella et al. (2024), the investigators assessed ongoing drug self-administration or yohimbine-induced reinstatement, a relapse-related measure we did not test in our study. As discussed elsewhere, results from many studies showed that the mechanisms of ongoing drug self-administration are different from those controlling relapse/reinstatement in animal models; there is also evidence that different mechanisms mediate the effects of different relapse-provoking stimuli (Shalev et al. 2002; Venniro et al. 2020).

As with AT-201, chronic delivery of J-113397 had no effect on any relapse-related measures in males. J-113397 also had a minimal effect on extinction, context-induced reinstatement, and reacquisition in females. However, our exploratory post-hoc statistical analysis suggests that J-113397 modestly decreased ‘incubated’ opioid seeking on day 8 (Fig. 3C). We also did not observe any clear opioid-like withdrawal symptoms after naloxone injections in rats implanted with minipumps containing J-113397, which is expected since J-113397 has very low affinity at MOR (Table 1 from Ozaki et al. 2000b), but see (Table 1 from Zaratin et al. 2004). Finally, an issue to consider with the largely negative results with both AT-201 and J-113397 is whether chronic minipump delivery resulted in pharmacologically effective doses in blood and brain.

Regarding J-113397, our mass spectrometry analysis showed reliable dose-dependent increases in plasma drug’s concentrations. Regarding AT-201, we did not perform mass spectrometry analysis, but observed robust naloxone-precipitated withdrawal signs, suggesting that under our experimental conditions, AT-201 acts at MORs like buprenorphine. Additionally, chronic AT-201 delivery *increased* heroin self-administration in females, further suggesting that we used effective pharmacological doses. In this regard, the chronic doses used here were based on effective acute doses of AT-201 and J-113397 (Recker and Higgins 2004; Khroyan et al. 2009; Kallupi et al. 2018). However, it is possible that a higher AT-201 dose would show an inhibitory effect on the relapse measures. Finally, another caveat is that we only tested two doses of each compound.

Another methodological consideration is that in our previous opioid maintenance validation study with buprenorphine, we used oxycodone as the self-administration drug whereas in the current study, we used heroin. Differences in opioid use severity and consequently success of buprenorphine treatment outcome between users of heroin compared with users of prescription opioid analgesics have been reported (Moore et al. 2007). The differences in the inhibitory effects of buprenorphine on relapse measures using an oxycodone versus heroin maintenance model have not been explored here but could underlie the differences seen in the lack of effects of AT-201 and BU08028 versus buprenorphine in our rat model. However, this possibility is unlikely, because in ongoing studies, we found that buprenorphine also inhibits the different relapse-related measures in rats with a history of heroin self-administration (Bossert JM. Unpublished).

### Sex differences in the effect of AT-201 and J-113397 on relapse-related behaviors

An unexpected finding was the somewhat sex-dependent effects of chronic AT-201 and J-113397 on the relapse-related measures. As mentioned above, the mixed NOP/MOR compounds had no effect in males. In contrast, AT-201 modestly increased reacquisition and J-113397 modestly decreased incubation in females. This potential sex-specific effects of the NOP compounds extend results from our previous reports using the opioid maintenance rat model with the MOR partial agonist TRV130 and BU08028 (Bossert et al. 2020, 2022). TRV130 decreased extinction responding in both sexes, but selectively decreased context-induced reinstatement and reacquisition of oxycodone self-administration in males (Bossert et al. 2020). BU08028 decreased incubation of heroin seeking in both sexes, selectively decreased extinction responding in males, and selectively increased reacquisition in females (Bossert et al. 2022).

These sex-specific behavioral effects may be due to sex differences in MOR and NOP receptor expression and function (Craft 2008; Zhang et al. 2012, 2018; Becker and Chartoff 2019). There is also evidence from antinociception studies that sex differences are more pronounced with low efficacy MOR agonists than with high efficacy MOR agonists (Cook et al. 2000). In this regard, pretreatment with low efficacy MOR partial agonists selectively decreased morphine analgesia in females but not males (Cook et al. 2000). This observation could potentially explain the increase in reacquisition of heroin self-administration with the NOP/MOR partial agonists BU08028 and AT-201, because at heroin unit doses on the descending dose–response curve, antagonism of MOR causes a compensatory increase in heroin self-administration (Yokel 1987). However, an argument against this possibility is that, as mentioned above, we found that that chronic buprenorphine (a low efficacy MOR partial agonist) decreases extinction responding, context-induced reinstatement, and reacquisition of oxycodone self-administration in both sexes (Bossert et al. 2020). Additionally, Bakhti-Suroosh et al. (2021) found that chronic buprenorphine decreases extinction responding and discrete cue-induced reinstatement of fentanyl seeking in both sexes.

Together, the results from the present study and our previous studies with oxycodone (Bossert et al. 2020; Fredriksson et al. 2020) highlight the importance of including both sexes in studies aimed at identifying novel medications for relapse prevention in animal models. This is because even under conditions where the relapse-related behavior is similar in males and females, the response to the target medications can be sex-specific (Fredriksson et al. 2021; Nicolas et al. 2022; Negishi et al. in press).

### Concluding remarks

We used a rat model of opioid maintenance to test the effect of chronic delivery of AT-201 and J-113397 on heroin relapse-related measures. In males, the NOP compounds had no effect on any of the relapse-related measures. In females, J-113397 had some potential beneficial effects (decreased incubation) whereas AT-201 had some potential detrimental effects (increased reacquisition). In our rat model of opioid agonist maintenance therapy, the mixed NOP/MOR partial agonists did not mimic buprenorphine’s inhibitory effects on the relapse-related measures. To the degree that the relapse-related measures used in our opioid maintenance model predict medication efficacy in humans (Epstein et al. 2006; Venniro et al. 2020), our results suggest that NOP/MOR partial agonists are unlikely to mimic the therapeutic effects of buprenorphine.

## Supplementary Information

Below is the link to the electronic supplementary material.

Supplementary file1 (DOCX 62 KB)
