# Supplementary material for: Effect of chronic delivery of the NOP/MOR partial agonist AT-201 and NOP antagonist J-113397 on heroin relapse in a rat model of opioid maintenance
Source: Psychopharmacology (Berl). 2024 Sep 13;241(12):2497–511. doi: 10.1007/s00213-024-06678-7 (PMC11569015; doi:10.1007/s00213-024-06678-7)
Supplement: Supplementary file 1 — Supplementary file1 (DOCX 62 KB) [file 213_2024_6678_MOESM1_ESM.docx]

**Supplemental Online Material**

8-19-2024 Psychopharmacology

Supplementary text

**Effect of chronic delivery of the NOP/MOR partial agonist AT-201 and NOP antagonist J-113397 on heroin relapse in a rat model of opioid maintenance**

Jennifer M. Bossert^1🖂^, Kiera E. Caldwell^1^, Hannah Korah^1^, Ashley Batista^1^, Hannah Bonbrest^1^, Ida Fredriksson^1^, Shelley N. Jackson^2^, Agnieszka Sulima^2^, Kenner C. Rice^2^, Nurulain T. Zaveri^3^, and Yavin Shaham^1^

.

**Table of content**

Table S1 (statistical reporting)

Table S2 Naloxone-precipitated withdrawal

Table S3 J-113397 Plasma measurements

**Table S1.** Statistical analysis for Experiments 1-3 (SPSS GLM repeated-measures module). Partial Eta^2^ = proportion of explained variance. NP, not possible to calculate partial Eta^2^. For ANCOVAs, the covariate is inactive lever presses. RM, repeated measures

**Exp. 1: Effect of AT-201 and J-113397 on food self-administration: male and female rats**

| **Figure number** | **Factor name** | **F-value** | ***p*-value** | **Partial Eta^2^** |
| --- | --- | --- | --- | --- |
| Figure 1B. AT-201  Food self-administration  Pellets  RM-ANOVA | AT-201 Dose (0, 12 mg/kg/d) between-subjects  Session (1-7) within-subjects  AT-201 Dose x Session | F_1,10_=0.8  F_6,60_=1.2  F_6,60_=0.7 | 0.405  0.298  0.613 | 0.070  0.111  0.070 |
| Figure 1B. AT-201  Food self-administration  Lever presses  RM-ANOVA | AT-201 Dose (0, 12 mg/kg/d) between-subjects  Lever (inactive) within-subjects  Lever x AT-201 Dose  Session (1-7) within-subjects  Session x AT-201 Dose  Lever x Session  Lever x Session x AT-201 Dose | F_1,10_=0.2  F_1,10_=99.5  F_1,10_=0.4  F_6,60_=1.7  F_6,60_=0.7  F_6,60_=1.8  F_6,60_=0.7 | 0.701  <0.001*  0.562  0.128  0.639  0.109  0.643 | 0.015  0.909  0.035  0.148  0.067  0.155  0.066 |
| Figure 1C. J-113397  Food self-administration  Pellets  RM-ANOVA | J-113397 Dose (0, 40 mg/kg/d) between-subjects  Session (1-7) within-subjects  J-113397 Dose x Session | F_1,11_=0.0  F_6,66_=14.6  F_6,66_=2.5 | 0.838  <0.001*  0.032* | 0.004  0.570  0.184 |
| Figure 1C. J-113397  Food self-administration  Lever presses  RM-ANOVA | J-113397 Dose (0, 40 mg/kg/d) between-subjects  Lever (inactive) within-subjects  Lever x J-113397 Dose  Session (1-7) within-subjects  Session x J-113397 Dose  Lever x Session  Lever x Session x J-113397 Dose | F_1,11_=0.1  F_1,11_=156.8  F_1,11_=0.0  F_6,66_=7.9  F_6,66_=1.9  F_6,66_=8.2  F_6,66_=2.5 | 0.769  <0.001*  0.962  <0.001*  0.100  <0.001*  0.033* | 0.008  0.934  0.000  0.419  0.145  0.428  0.182 |

**Exp. 2 & 3: Heroin self-administration training**

| **Figure number** | **Factor name** | **F-value** | ***p*-value** | **Partial Eta^2^** |
| --- | --- | --- | --- | --- |
| Figure 2B. AT-201 Heroin self-administration training (**males and females**)  Infusions  RM-ANOVA | Sex (M, F) between-subjects  Heroin Dose (0.1, 0.05 mg/kg/infusion) within-subjects  Heroin Dose x Sex  Session (1-7, 8-14) within-subjects  Session x Sex  Heroin Dose x Session  Heroin Dose x Session x Sex | F_1,51_=1.2  F_1,51_=121.0  F_1,51_=0.0  F_6,306_=20.0  F_6,306_=0.6  F_6,306_=1.5  F_6,306_=1.1 | 0.270  <0.001*  0.997  <0.001*  0.764  0.193  0.393 | 0.024  0.703  0.000  0.281  0.011  0.028  0.020 |
| Figure 2B. AT-201 Heroin self-administration training (**males**)  Infusions  RM-ANOVA | Heroin Dose (0.1, 0.05 mg/kg/infusion) within-subjects  Session (1-7, 8-14) within-subjects  Heroin Dose x Session | F_1,23_=47.2  F_6,138_=8.5  F_6,138_=1.6 | <0.001*  <0.001*  0.154 | 0.673  0.270  0.065 |
| Figure 2B. AT-201 Heroin self-administration training (**females**)  Infusions  RM-ANOVA | Heroin Dose (0.1, 0.05 mg/kg/infusion) within-subjects  Session (1-7, 8-14) within-subjects  Heroin Dose x Session | F_1,28_=77.6  F_6,168_=12.4  F_6,168_=0.5 | <0.001*  <0.001*  0.829 | 0.735  0.307  0.017 |
| Figure 2B. AT-201 Heroin self-administration training (**males and females**)  Lever presses  RM-ANOVA | Sex (M, F) between-subjects  Lever (inactive, active)  Lever x Sex  Heroin Dose (0.1, 0.05 mg/kg/infusion) within-subjects  Heroin Dose x Sex  Session (1-7, 8-14) within-subjects  Session x Sex  Lever x Heroin Dose  Lever x Heroin Dose x Sex  Lever x Session  Lever x Session x Sex  Heroin Dose x Session  Heroin Dose x Session x Sex  Lever x Heroin Dose x Session  Lever x Heroin Dose x Session x Sex | F_1,51_=0.1  F_1,51_=36.9  F_1,51_=0.1  F_1,51_=19.1  F_1,51_=0.8  F_6,306_=1.6  F_6,306_=1.7  F_1,51_=17.4  F_1,51_=0.8  F_6,306_=3.1  F_6,306_=1.5  F_6,306_=1.1  F_6,306_=1.2  F_6,306_=1.3  F_6,306_=1.3 | 0.740  <0.001*  0.791  <0.001*  0.379  0.152  0.120  <0.001*  0.367  0.006*  0.176  0.391  0.309  0.277  0.246 | 0.002  0.420  0.001  0.273  0.015  0.030  0.032  0.254  0.016  0.058  0.029  0.020  0.023  0.024  0.025 |
| Figure 2B. AT-201 Heroin self-administration training (**males**)  Lever presses  RM-ANOVA | Lever (inactive, active)  Heroin Dose (0.1, 0.05 mg/kg/infusion) within-subjects  Session (1-7, 8-14) within-subjects  Lever x Heroin Dose  Lever x Session  Heroin Dose x Session  Lever x Heroin Dose x Session | F_1,23_=9.5  F_1,23_=6.1  F_6,138_=0.7  F_1,23_=5.7  F_6,138_=0.9  F_6,138_=1.0  F_6,138_=1.1 | 0.005*  0.021*  0.685  0.026*  0.491  0.451  0.352 | 0.292  0.211  0.028  0.198  0.038  0.040  0.047 |
| Figure 2B. AT-201 Heroin self-administration training (**females**)  Lever presses  RM-ANOVA | Lever (inactive, active)  Heroin Dose (0.1, 0.05 mg/kg/infusion) within-subjects  Session (1-7, 8-14) within-subjects  Lever x Heroin Dose  Lever x Session  Heroin Dose x Session  Lever x Heroin Dose x Session | F_1,28_=82.0  F_1,28_=52.7  F_6,168_=6.4  F_1,28_=49.4  F_6,168_=8.9  F_6,168_=0.5  F_6,168_=0.4 | <0.001*  <0.001*  <0.001*  <0.001*  <0.001*  0.778  0.901 | 0.745  0.653  0.185  0.638  0.242  0.019  0.013 |
| Figure 2C. J-113397 Heroin self-administration training (**males and females**)  Infusions  RM-ANOVA | Sex (M, F) between-subjects  Heroin Dose (0.1, 0.05 mg/kg/infusion) within-subjects  Heroin Dose x Sex  Session (1-7, 8-14) within-subjects  Session x Sex  Heroin Dose x Session  Heroin Dose x Session x Sex | F_1,52_=3.5  F_1,52_=177.4  F_1,52_=0.3  F_6,312_=15.3  F_6,312_=1.2  F_6,312_=3.2  F_6,312_=0.5 | 0.065  <0.001*  0.601  <0.001*  0.288  0.005*  0.819 | 0.064  0.773  0.005  0.228  0.023  0.058  0.009 |
| Figure 2C. J-113397 Heroin self-administration training (**males**)  Infusions  RM-ANOVA | Heroin Dose (0.1, 0.05 mg/kg/infusion) within-subjects  Session (1-7, 8-14) within-subjects  Heroin Dose x Session | F_1,22_=118.1  F_6,132_=7.8  F_6,132_=2.0 | <0.001*  <0.001*  0.069 | 0.843  0.261  0.084 |
| Figure 2C. J-113397 Heroin self-administration training (**females**)  Infusions  RM-ANOVA | Heroin Dose (0.1, 0.05 mg/kg/infusion) within-subjects  Session (1-7, 8-14) within-subjects  Heroin Dose x Session | F_1,30_=89.8  F_6,180_=10.4  F_6,180_=2.1 | <0.001*  <0.001*  0.051 | 0.750  0.258  0.067 |
| Figure 2C. J-113397 Heroin self-administration training (**males and females**)  Lever presses  RM-ANOVA | Sex (M, F) between-subjects  Lever (inactive, active)  Lever x Sex  Heroin Dose (0.1, 0.05 mg/kg/infusion) within-subjects  Heroin Dose x Sex  Session (1-7, 8-14) within-subjects  Session x Sex  Lever x Heroin Dose  Lever x Heroin Dose x Sex  Lever x Session  Lever x Session x Sex  Heroin Dose x Session  Heroin Dose x Session x Sex  Lever x Heroin Dose x Session  Lever x Heroin Dose x Session x Sex | F_1,52_=0.3  F_1,52_=307.7  F_1,52_=0.9  F_1,52_=97.1  F_1,52_=0.2  F_6,312_=7.1  F_6,312_=1.4  F_1,52_=83.4  F_1,52_=0.6  F_6,312_=14.2  F_6,312_=0.8  F_6,312_=1.0  F_6,312_=0.8  F_6,312_=3.1  F_6,312_=0.9 | 0.567  <0.001*  0.342  <0.001*  0.686  <0.001*  0.221  <0.001*  0.438  <0.001*  0.576  0.444  0.581  0.006*  0.519 | 0.006  0.855  0.017  0.637  0.003  0.120  0.026  0.616  0.012  0.215  0.015  0.018  0.015  0.056  0.016 |
| Figure 2C. J-113397 Heroin self-administration training (**males**)  Lever presses  RM-ANOVA | Lever (inactive, active)  Heroin Dose (0.1, 0.05 mg/kg/infusion) within-subjects  Session (1-7, 8-14) within-subjects  Lever x Heroin Dose  Lever x Session  Heroin Dose x Session  Lever x Heroin Dose x Session | F_1,22_=192.1  F_1,22_=41.5  F_6,132_=2.2  F_1,22_=34.6  F_6,132_=5.1  F_6,132_=0.8  F_6,132_=2.2 | <0.001*  <0.001*  0.045*  <0.001*  <0.001*  0.549  0.051 | 0.897  0.653  0.092  0.611  0.188  0.036  0.089 |
| Figure 2C. J-113397 Heroin self-administration training (**females**)  Lever presses  RM-ANOVA | Lever (inactive, active)  Heroin Dose (0.1, 0.05 mg/kg/infusion) within-subjects  Session (1-7, 8-14) within-subjects  Lever x Heroin Dose  Lever x Session  Heroin Dose x Session  Lever x Heroin Dose x Session | F_1,30_=157.4  F_1,30_=53.4  F_6,180_=6.9  F_1,30_=53.0  F_6,180_=10.8  F_6,180_=1.0  F_6,180_=1.9 | <0.001*  <0.001*  <0.001*  <0.001*  <0.001*  0.400  0.083 | 0.840  0.640  0.187  0.639  0.265  0.034  0.060 |

**Exp. 2 & 3: Effect of chronic AT-201 and J-113397 on incubation of heroin seeking in Context B**

| **Figure number** | **Factor name** | **F-value** | ***p*-value** | **Partial Eta^2^** |
| --- | --- | --- | --- | --- |
| Figure 3B. AT-201 Incubation of heroin seeking (**males and females**)  Lever presses  RM-ANOVA | Sex (M, F) between-subjects  AT-201 Dose (0, 3.8, 12 mg/kg/d) between-subjects  Sex x AT-201 Dose  Lever (inactive, active) within-subjects Lever x Sex  Lever x AT-201 Dose  Lever x Sex x AT-201 Dose  Day (1, 8) within-subjects  Day x Sex  Day x AT-201 Dose  Day x Sex x AT-201 Dose  Lever x Day  Lever x Day x Sex  Lever x Day x AT-201 Dose  Lever x Day x Sex x AT-201 Dose | F_1,47_=1.3  F_2,47_=0.7  F_2,47_=0.2  F_1,47_=103.5  F_1,47_=2.6  F_2,47_=0.5  F_2,47_=0.3  F_1,47_=13.4  F_1,47_=0.4  F_2,47_=1.9  F_2,47_=1.1  F_1,47_=12.7  F_1,47_=0.0  F_2,47_=1.6  F_2,47_=0.6 | 0.260  0.520  0.788  <0.001*  0.117  0.638  0.744  <0.001*  0.532  0.158  0.339  0.001*  0.838  0.217  0.567 | 0.027  0.027  0.010  0.688  0.052  0.019  0.013  0.222  0.008  0.075  0.045  0.213  0.001  0.063  0.024 |
| Figure 3B. AT-201 Incubation of heroin seeking (**males**)  Lever presses  RM-ANOVA | AT-201 Dose (0, 3.8, 12 mg/kg/d) between-subjects  Lever (inactive, active) within-subjects  Lever x AT-201 Dose  Day (1, 8) within-subjects  Day x AT-201 Dose  Lever x Day  Lever x Day x AT-201 Dose | F_2,21_=0.4  F_1,21_=106.7  F_2,21_=0.8  F_1,21_=12.3  F_2,21_=1.1  F_1,21_=17.2  F_2,21_=0.5 | 0.648  <0.001*  0.452 0.002* 0.359 <0.001*  0.600 | 0.041  0.836  0.073  0.370  0.093  0.450  0.047 |
| Figure 3B. AT-201 Incubation of heroin seeking (**females**)  Lever presses  RM-ANOVA | AT-201 Dose (0, 3.8, 12 mg/kg/d) between-subjects  Lever (inactive, active) within-subjects  Lever x AT-201 Dose  Day (1, 8) within-subjects  Day x AT-201 Dose  Lever x Day  Lever x Day x AT-201 Dose | F_2,26_=0.5  F_1,26_=49.2  F_2,26_=0.3  F_1,26_=6.6  F_2,26_=1.9  F_1,26_=5.0  F_2,26_=1.4 | 0.591  <0.001*  0.714  0.016* 0.170 0.034* 0.263 | 0.040  0.654  0.026  0.203  0.127  0.162  0.098 |
| Figure 3C. J-113397 Incubation of heroin seeking (**males and females**)  Lever presses  RM-ANOVA | Sex (M, F) between-subjects  J-113397 Dose (0, 12.6, 40 mg/kg/d) between-subjects  Sex x J-113397 Dose  Lever (inactive, active)  Lever x Sex  Lever x J-113397 Dose  Lever x Sex x J-113397 Dose  Day (1, 8) within-subjects  Day x Sex  Day x J-113397 Dose  Day x Sex x J-113397 Dose  Lever x Day  Lever x Day x Sex  Lever x Day x J-113397 Dose  Lever x Day x Sex x J-113397 Dose | F_1,47_=0.2  F_2,47_=1.0  F_2,47_=2.0  F_1,47_=176.1  F_1,47_=0.0  F_2,47_=0.2  F_2,47_=1.1  F_1,47_=3.8  F_1,47_=1.2  F_2,47_=1.2  F_2,47_=3.3  F_1,47_=2.9  F_1,47_=3.0  F_2,47_=0.5  F_2,47_=3.1 | 0.666  0.389  0.149  <0.001*  0.909  0.824  0.332  0.057  0.283  0.300  0.046*  0.094  0.088  0.591  0.053 | 0.004  0.039  0.078  0.789  0.000  0.008  0.046  0.075  0.024  0.050  0.123  0.059  0.061  0.022  0.117 |
| Figure 3C. J-113397 Incubation of heroin seeking (**males**)  Lever presses  RM-ANOVA | J-113397 Dose (0, 12.6, 40 mg/kg/d) between-subjects  Lever (inactive, active)  Lever x J-113397 Dose  Day (1, 8) within-subjects  Day x J-113397 Dose  Day x Lever  Day x Lever x J-113397 Dose | F_2,20_=1.2  F_1,20_=134.4  F_2,20_=0.6  F_1,20_=12.6  F_2,20_=0.7  F_1,20_=12.6  F_2,20_=1.2 | 0.326  <0.001*  0.583  0.002*  0.506  0.002*  0.334 | 0.106  0.871  0.053  0.386  0.066  0.387  0.104 |
| Figure 3C. J-113397 Incubation of heroin seeking (**females**)  Lever presses  RM-ANOVA | J-113397 Dose (0, 12.6, 40 mg/kg/d) between-subjects  Lever (inactive, active)  Lever x J-113397 Dose  Day (1, 8) within-subjects  Day x J-113397 Dose  Day x Lever  Day x Lever x J-113397 Dose | F_2,27_=1.9  F_1,27_=78.3  F_2,27_=0.8  F_1,27_=0.3  F_2,27_=3.2  F_1,27_=0.0  F_2,27_=2.5 | 0.176  <0.001*  0.448  0.596  0.055  0.984  0.104 | 0.121  0.744  0.058  0.011  0.193  0.0002  0.155 |

**Exp. 2 & 3: Effect of AT-201 and J-113397 on extinction responding in Context B**

| **Figure number** | **Factor name** | **F-value** | ***p*-value** | **Partial Eta^2^** |
| --- | --- | --- | --- | --- |
| Figure 4B. AT-201 Extinction (**males and females**)  Lever presses  RM-ANOVA | Sex (M, F) between-subjects  AT-201 Dose (0, 3.8, 12 mg/kg/d) between-subjects  Sex x AT-201 Dose  Lever (inactive, active) within-subjects  Lever x Sex  Lever x AT-201 Dose  Lever x Sex x AT-201 Dose  Session (1-7) within-subjects  Session x Sex  Session x AT-201 Dose  Session x Sex x AT-201 Dose  Lever x Session  Lever x Session x Sex  Lever x Session x AT-201 Dose  Lever x Session x Sex x AT-201 Dose | F_1,47_=1.2  F_2,47_=0.0  F_2,47_=1.9  F_1,47_=129.2  F_1,47_=5.1  F_2,47_=0.2  F_2,47_=0.6  F_6,282_=68.9  F_6,282_=1.1  F_12,282_=1.2  F_12,282_=0.3  F_6,282_=54.9  F_6,282_=1.9  F_12,282_=0.6  F_12,282_=0.5 | 0.277  0.983  0.161  <0.001*  0.028*  0.808  0.533  <0.001*  0.373  0.316  0.981  <0.001*  0.087  0.876  0.942 | 0.025  0.001  0.075  0.733  0.098  0.009  0.026  0.594  0.023  0.047  0.014  0.539  0.038  0.023  0.019 |
| Figure 4B. AT-201 Extinction (**males**)  Lever presses  RM-ANOVA | AT-201 Dose (0, 3.8, 12 mg/kg/d) between-subjects  Lever (inactive, active) within-subjects  Lever x AT-201 Dose  Session (1-7) within-subjects  Session x AT-201 Dose  Lever x Session  Lever x Session x AT-201 Dose | F_2,21_=1.7  F_1,21_=140.2  F_2,21_=1.7  F_6,126_=49.1  F_12,126_=1.9  F_6,126_=39.3  F_12,126_=1.4 | 0.213  <0.001*  0.199 <0.001*  0.037*  <0.001*  0.179 | 0.137  0.870  0.142  0.700  0.155  0.652  0.117 |
| Figure 4B. AT-201 Extinction (**females**)  Lever presses  RM-ANOVA | AT-201 Dose (0, 3.8, 12 mg/kg/d) between-subjects  Lever (inactive, active) within-subjects  Lever x AT-201 Dose  Session (1-7) within-subjects  Session x AT-201 Dose  Lever x Session  Lever x Session x AT-201 Dose | F_2,26_=0.8  F_1,26_=64.4  F_2,26_=0.2  F_6,156_=33.6  F_12,156_=0.3  F_6,156_=28.9  F_12,156_=0.3 | 0.461  <0.001*  0.797  <0.001*  0.981  <0.001*  0.993 | 0.058  0.713  0.017  0.564  0.025  0.526  0.020 |
| Figure 4C. J-113397 Extinction (**males and females**)  Lever presses  RM-ANOVA | Sex (M, F) between-subjects  J-113397 Dose (0, 12.6, 40 mg/kg/d) between-subjects  Sex x J-113397 Dose  Lever (inactive, active) within-subjects  Lever x Sex  Lever x J-113397 Dose  Lever x Sex x J-113397 Dose  Session (1-7) within-subjects  Session x Sex  Session x J-113397 Dose  Session x Sex x J-113397 Dose  Lever x Session  Lever x Session x Sex  Lever x Session x J-113397 Dose  Lever x Session x Sex x J-113397 Dose | F_1,48_=0.1  F_2,48_=0.5  F_2,48_=0.6  F_1,48_=240.1  F_1,48_=0.0  F_2,48_=1.3  F_2,48_=0.8  F_6,288_=50.1  F_6,288_=0.6  F_12,288_=1.4  F_12,288_=0.7  F_6,288_=54.6  F_6,288_=0.6  F_12,288_=1.0  F_12,288_=0.4 | 0.806  0.637  0.560  <0.001*  0.886  0.283  0.441  <0.001*  0.711  0.162  0.714  <0.001*  0.747  0.480  0.955 | 0.001  0.019  0.024  0.833  0.000  0.051  0.033  0.511  0.013  0.055  0.030  0.532  0.012  0.039  0.017 |
| Figure 4C. J-113397 Extinction (**males**)  Lever presses  RM-ANOVA | J-113397 Dose (0, 12.6, 40 mg/kg/d) between-subjects  Lever (inactive, active) within-subjects  Lever x J-113397 Dose  Session (1-7) within-subjects  Session x J-113397 Dose  Lever x Session  Lever x Session x J-113397 Dose | F_2,20_=0.6  F_1,20_=219.1  F_2,20_=1.7  F_6,120_=69.1  F_12,120_=1.4  F_6,120_=62.7  F_12,120_=1.0 | 0.556  <0.001*  0.203  <0.001*  0.171  <0.001*  0.479 | 0.057  0.916  0.147  0.776  0.123  0.758  0.089 |
| Figure 4C. J-113397 Extinction (**females**)  Lever presses  RM-ANOVA | J-113397 Dose (0, 12.6, 40 mg/kg/d) between-subjects  Lever (inactive, active) within-subjects  Lever x J-113397 Dose  Session (1-7) within-subjects  Session x J-113397 Dose  Lever x Session  Lever x Session x J-113397 Dose | F_2,28_=0.6  F_1,28_=104.1  F_2,28_=1.1  F_6,28_=19.1  F_12,168_=1.3  F_6,168_=20.7  F_12,168_=0.8 | 0.542  <0.001*  0.361  <0.001*  0.252  <0.001*  0.700 | 0.043  0.788  0.070  0.406  0.082  0.425  0.051 |

**Exp. 2 & 3: Effect of AT-201 and J-113397 on context-induced reinstatement**

| **Figure number** | **Factor name** | **F-value** | ***p*-value** | **Partial Eta^2^** |
| --- | --- | --- | --- | --- |
| Figure 5B. AT-201 Context-induced reinstatement (**males and females**)  Lever presses  RM-ANOVA | Sex (M, F) between-subjects  AT-201 Dose (0, 3.8, 12 mg/kg/d) between-subjects  Sex x AT-201 Dose  Lever (inactive, active) within-subjects  Lever x Sex  Lever x AT-201 Dose  Lever x Sex x AT-201 Dose  Context (B, A) within-subjects  Context x Sex  Context x AT-201 Dose  Context x Sex x AT-201 Dose  Lever x Context  Lever x Context x Sex  Lever x Context x AT-201 Dose  Lever x Context x Sex x AT-201 Dose | F_1,46_=1.1  F_2,46_=0.2  F_2,46_=0.6  F_1,46_=126.2  F_1,46_=2.6  F_2,46_=0.2  F_2,46_=0.8  F_1,46_=70.0  F_1,46_=0.7  F_2,46_=0.3  F_2,46_=0.9  F_1,46_=64.5  F_1,46_=1.0  F_2,46_=0.4  F_2,46_=0.7 | 0.294  0.813  0.570  <0.001*  0.114  0.860  0.450  <0.001*  0.395  0.743  0.433  <0.001*  0.324  0.674  0.513 | 0.024  0.009  0.024  0.733  0.053  0.007  0.034  0.603  0.016  0.013  0.036  0.584  0.021  0.017  0.029 |
| Figure 5B. AT-201 Context-induced reinstatement (**males**)  Lever presses  RM-ANOVA | AT-201 Dose (0, 3.8, 12 mg/kg/d) between-subjects  Lever (inactive, active) within-subjects  Lever x AT-201 Dose  Context (B, A) within-subjects  Context x AT-201 Dose  Lever x Context  Lever x Context x AT-201 Dose | F_2,20_=0.1  F_1,20_=50.2  F_2,20_=0.4  F_1,20_=23.7  F_2,20_=0.2  F_1,20_=25.9  F_2,20_=0.2 | 0.899  <0.001*  0.656  <0.001*  0.812  <0.001*  0.817 | 0.011  0.715  0.041  0.542  0.021  0.564  0.020 |
| Figure 5B. AT-201 Context-induced reinstatement (**females**)  Lever presses  RM-ANOVA | AT-201 Dose (0, 3.8, 12 mg/kg/d) between-subjects  Lever (inactive, active) within-subjects  Lever x AT-201 Dose  Context (B, A) within-subjects  Context x AT-201 Dose  Lever x Context  Lever x Context x AT-201 Dose | F_2,26_=0.6  F_1,26_=82.4  F_2,26_=0.5  F_1,26_=50.6  F_2,26_=1.0  F_1,26_=41.5  F_2,26_=0.9 | 0.534  <0.001*  0.586  <0.001*  0.369  <0.001*  0.434 | 0.047  0.760  0.040  0.661  0.074  0.615  0.062 |
| Figure 5C. J-113397 Context-induced reinstatement (**males and females**)  Lever presses  RM-ANOVA | Sex (M, F) between-subjects  J-113397 Dose (0, 12.6, 40 mg/kg/d) between-subjects  Sex x J-113397 Dose  Lever (inactive, active) within-subjects  Lever x Sex  Lever x J-113397 Dose  Lever x Sex x J-113397 Dose  Context (B, A) within-subjects  Context x Sex  Context x J-113397 Dose  Context x Sex x J-113397 Dose  Lever x Context  Lever x Context x Sex  Lever x Context x J-113397 Dose  Lever x Context x Sex x J-113397 Dose | F_1,48_=0.4  F_2,48_=2.0  F_2,48_=1.1  F_1,48_=198.5  F_1,48_=0.3  F_2,48_=0.7  F_2,48_=0.5  F_1,48_=88.5  F_1,48_=0.8  F_2,48_=2.0  F_2,48_=1.6  F_1,48_=64.6  F_1,48_=1.2  F_2,48_=1.6  F_2,48_=0.7 | 0.530  0.145  0.356  <0.001*  0.580  0.495  0.599  <0.001*  0.379  0.150  0.220  <0.001*  0.282  0.207  0.480 | 0.008  0.077  0.042  0.805  0.006  0.029  0.021  0.648  0.016  0.076  0.061  0.574  0.024  0.064  0.030 |
| Figure 5C. J-113397 Context-induced reinstatement (**males**)  Lever presses  RM-ANOVA | J-113397 Dose (0, 12.6, 40 mg/kg/d) between-subjects  Lever (inactive, active) within-subjects  Lever x J-113397 Dose  Context (B, A) within-subjects  Context x J-113397 Dose  Lever x Context  Lever x Context x J-113397 Dose | F_2,20_=0.3  F_1,20_=137.0  F_2,20_=0.4  F_1,20_=36.5  F_2,20_=1.9  F_1,20_=28.6  F_2,20_=1.5 | 0.754  <0.001*  0.687  <0.001*  0.179  <0.001*  0.245 | 0.028  0.873  0.037  0.646  0.158  0.588  0.131 |
| Figure 5C. J-113397 Context-induced reinstatement (**females**)  Lever presses  RM-ANOVA | J-113397 Dose (0, 12.6, 40 mg/kg/d) between-subjects  Lever (inactive, active) within-subjects  Lever x J-113397 Dose  Context (B, A) within-subjects  Context x J-113397 Dose  Lever x Context  Lever x Context x J-113397 Dose | F_2,28_=3.0  F_1,28_=87.7  F_2,28_=0.9  F_1,28_=56.8  F_2,28_=1.7  F_1,28_=41.2  F_2,28_=1.1 | 0.065  <0.001*  0.418  <0.001*  0.193  <0.001*  0.358 | 0.178  0.758  0.060  0.670  0.111  0.595  0.071 |

**Exp. 2 & 3: Effect of AT-201 and J-113397 on reacquisition in Context A**

| **Figure number** | **Factor name** | **F-value** | ***p*-value** | **Partial Eta^2^** |
| --- | --- | --- | --- | --- |
| Figure 6B. AT-201 Reacquisition (**males and females**)  Infusions  RM-ANOVA | Sex (M, F) between-subjects  AT-201 Dose (0, 3.8, 12 mg/kg/d) between-subjects  Sex x AT-201 Dose  Hour (1-6) within-subjects  Hour x Sex  Hour x AT-201 Dose  Hour x Sex x AT-201 Dose | F_1,47_=4.4  F_2,47_=3.6  F_2,47_=0.6  F_5,235_=2.3  F_5,235_=0.9  F_10,235_=1.3  F_10,235_=0.4 | 0.041*  0.034*  0.568  0.043*  0.471  0.220  0.944 | 0.086  0.134  0.024  0.047  0.019  0.053  0.017 |
| Figure 6B. AT-201 Reacquisition (**males**)  Infusions  RM-ANOVA | AT-201 Dose (0, 3.8, 12 mg/kg/d) between-subjects  Hour (1-6) within-subjects  Hour x AT-201 Dose | F_2,21_=0.9  F_5,105_=0.8  F_10,105_=0.7 | 0.429  0.554  0.688 | 0.077  0.037  0.066 |
| Figure 6B. AT-201 Reacquisition (**females**)  Infusions  RM-ANOVA | AT-201 Dose (0, 3.8, 12 mg/kg/d) between-subjects  Hour (1-6) within-subjects  Hour x AT-201 Dose | F_2,26_=3.2  F_5,130_=3.2  F_10,130_=1.0 | 0.057  0.010*  0.475 | 0.197  0.108  0.069 |
| Figure 6C. J-113397 Reacquisition (**males and females**)  Infusions  RM-ANOVA | Sex (M, F) between-subjects  J-113397 Dose (0, 12.6, 40 mg/kg/d) between-subject Sex x J-113397 Dose  Hour (1-6) within-subjects  Hour x Sex  Hour x J-113397 Dose  Hour x Sex x J-113397 Dose | F_1,44_=2.5  F_2,44_=1.1  F_2,44_=1.3  F_5,220_=7.2  F_5,220_=0.4  F_10,220_=1.7  F_10,220_=0.8 | 0.120  0.346  0.279  <0.001*  0.879  0.079  0.593 | 0.054  0.047  0.056  0.140  0.008  0.072  0.037 |
| Figure 6C. J-113397 Reacquisition (**males**)  Infusions  RM-ANOVA | J-113397 Dose (0, 12.6, 40 mg/kg/d) between-subjects  Hour (1-6) within-subjects  Hour x J-113397 Dose | F_2,20_=0.3  F_5,100_=5.1  F_10,100_=1.2 | 0.773  <0.001*  0.315 | 0.025  0.203  0.105 |
| Figure 6C. J-113397 Reacquisition (**females**)  Infusions  RM-ANOVA | J-113397 Dose (0, 12.6, 40 mg/kg/d) between-subjects  Hour (1-6) within-subjects  Hour x J-113397 Dose | F_2,24_=2.0  F_5,120_=3.1  F_10,120_=1.5 | 0.159  0.012*  0.167 | 0.142  0.113  0.108 |

**Table S2: Naloxone-precipitated withdrawal**

| **Compound** | **Baseline withdrawal score after saline injections (mean±SEM)** | **Naloxone withdrawal score (mean±SEM)** | **t-value** | **p-value** | **Body weight loss (Baseline weight – weight after naloxone (g))** |
| --- | --- | --- | --- | --- | --- |
| **AT-201** | Total (n=10): 6.1±1.5 | Total (n=10): 26.4±3.9 | t_9_=4.7 | 0.001* | 7.6±0.9 gram |
| **J-113397** | Total (n=9): 0.7±0.4 | Total (n=9): 9.0±1.8 | t_8_=1.0 | 0.347 | 0.3±0.5 gram |

**Table S3: J-113397 Plasma measurements**

| **Group** | **Total (n)** | **Concentration of J-113397 in Plasma (mean±SEM) in ng/mL** |
| --- | --- | --- |
| Vehicle | 4 | Not Detected |
| 10 mg/kg/day | 2 | 16.6±2.5 |
| 20 mg/kg/day | 2 | 85.6±5.6 |
| 40 mg/kg/day | 2 | 135.8±58.2 |
